# A Novel Technique of Overtube‐assisted Ultrathin Endoscopic Biliary Drainage Using Multi‐Hole Self‐Expandable Metal Stents: A Case Report

**DOI:** 10.1002/deo2.70253

**Published:** 2025-11-26

**Authors:** Akinobu Koiwai, Morihisa Hirota, Kei Ishikawa, Chihiro Yunomura, Takuro Nakaya, Yuki Miyashita, Nana Inomata, Kennichi Satoh

**Affiliations:** ^1^ Division of Gastroenterology Tohoku Medical and Pharmaceutical University Sendai City Japan

**Keywords:** biliary drainage, ERCP, overtube, self‐expandable metal stent, ultrathin endoscope

## Abstract

Endoscopic retrograde cholangiopancreatography (ERCP) is the standard procedure for biliary drainage; however, access can be challenging in patients with altered anatomy or tumor‐related distortion. Endoscopic ultrasound‐guided biliary drainage (EUS‐BD) is a common alternative, yet it is not always feasible, particularly in patients with prior hepatic resection or severe gastrointestinal deformation. A 67‐year‐old woman developed obstructive jaundice due to hilar biliary stricture secondary to peritoneal dissemination following colorectal cancer surgery. Initial ERCP achieved side‐by‐side placement of multi‐hole self‐expandable metal stents (MHSEMSs), resulting in effective drainage. At recurrence of cholangitis, repeat ERCP failed because of anatomical distortion, and EUS‐BD was not feasible due to the patient's prior left hepatectomy. We therefore employed a novel technique: a double‐balloon enteroscope overtube was advanced to the duodenum, and a side hole was created 10 cm distal to the insertion port. An ultrathin endoscope was inserted through this side hole, retroflexed in the duodenum, and successfully accessed the papilla. The previously placed MHSEMSs were removed, and new MHSEMSs were deployed using a stent‐in‐stent method. The patient's cholangitis and associated liver abscesses improved, allowing continuation of systemic chemotherapy. This case demonstrates a novel overtube‐assisted ultrathin endoscope technique. The combination of thin delivery systems and an overtube modification may provide a valuable alternative when both conventional ERCP and EUS‐BD are not feasible.

## 1 Introduction

Endoscopic retrograde cholangiopancreatography (ERCP) is the gold standard for biliary drainage in patients with malignant biliary obstruction. In most cases, standard duodenoscope access to the duodenal papilla is feasible. However, prior surgery, adhesions, or tumor‐related distortion may impede scope advancement. In such cases, endoscopic ultrasound‐guided biliary drainage (EUS‐BD) has emerged as a reliable alternative [1, 2]. Nevertheless, specific situations, including prior hepatectomy or the absence of suitable puncture routes, may preclude EUS‐BD. In such cases, percutaneous transhepatic biliary drainage (PTBD) remains the last resort but is invasive and often causes discomfort from external drainage. We present a unique case in which ultrathin endoscopy was combined with a double‐balloon enteroscope (DBE) overtube modified with a side hole, representing, to our knowledge, the first report of overtube‐assisted ultrathin endoscope ERCP with multi‐hole self‐expandable metal stents (MHSEMSs).

## 2 Case Report

**FIGURE 1:**
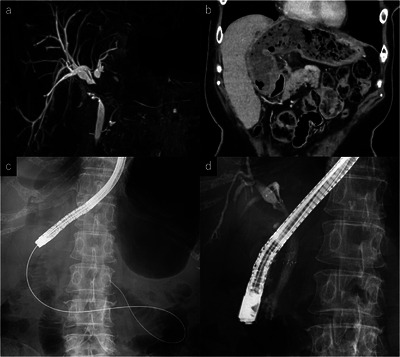
(a) MRCP showing hilar biliary stricture (Bismuth II) due to peritoneal dissemination, with dilatation of the intrahepatic bile ducts. MRCP: magnetic resonance cholangiopancreatography. (b) CECT demonstrated gastric deformation, with the distal stomach adherent to the remnant liver. CECT: contrast‐enhanced computed tomography. (c) Because of gastric deformation, a standard duodenoscope could not be advanced into the second portion of the duodenum. A forward‐viewing scope was used to insert a guidewire into the distal duodenum, and the duodenoscope was advanced over the wire. (d) Side‐by‐side placement of MHSEMSs into the right anterior and posterior segmental bile ducts for hilar biliary stricture. MHSEMSs: multi‐hole self‐expandable metal stents.

A 67‐year‐old woman with a history of rectal cancer underwent laparoscopic low anterior resection, followed by left hepatectomy for liver metastasis. She had been receiving systemic chemotherapy. During treatment, she developed perihilar biliary obstruction (Bismuth II) with obstructive jaundice and was referred to our department. Contrast‐enhanced computed tomography (CECT) and magnetic resonance cholangiopancreatography (MRCP) revealed intrahepatic bile duct dilatation caused by peritoneal dissemination (Figure 1a), and gastric deformation, with the distal stomach adherent to the remnant liver (Figure 1b).

First intervention: Because of gastric deformation, insertion of a standard duodenoscope (TJF‐Q290V; Olympus, Tokyo, Japan) was unsuccessful. A forward‐viewing scope (GIF‐EZ1500; Olympus) was used to insert a 0.045‐inch guidewire (Denis, Cardinal Health) into the distal duodenum (Figure 1c), and the duodenoscope was advanced over the wire. Biliary cannulation was achieved, and two MHSEMSs (HANAROSTENT Biliary Multi Hole Benefit; Boston Scientific, MA, USA, Figure S1a) were placed side‐by‐side across the papilla (right anterior segmental bile duct: 6 mm×8 cm, right posterior: 6 mm×10 cm) (Figure 1d), resulting in effective drainage.

**FIGURE 2:**
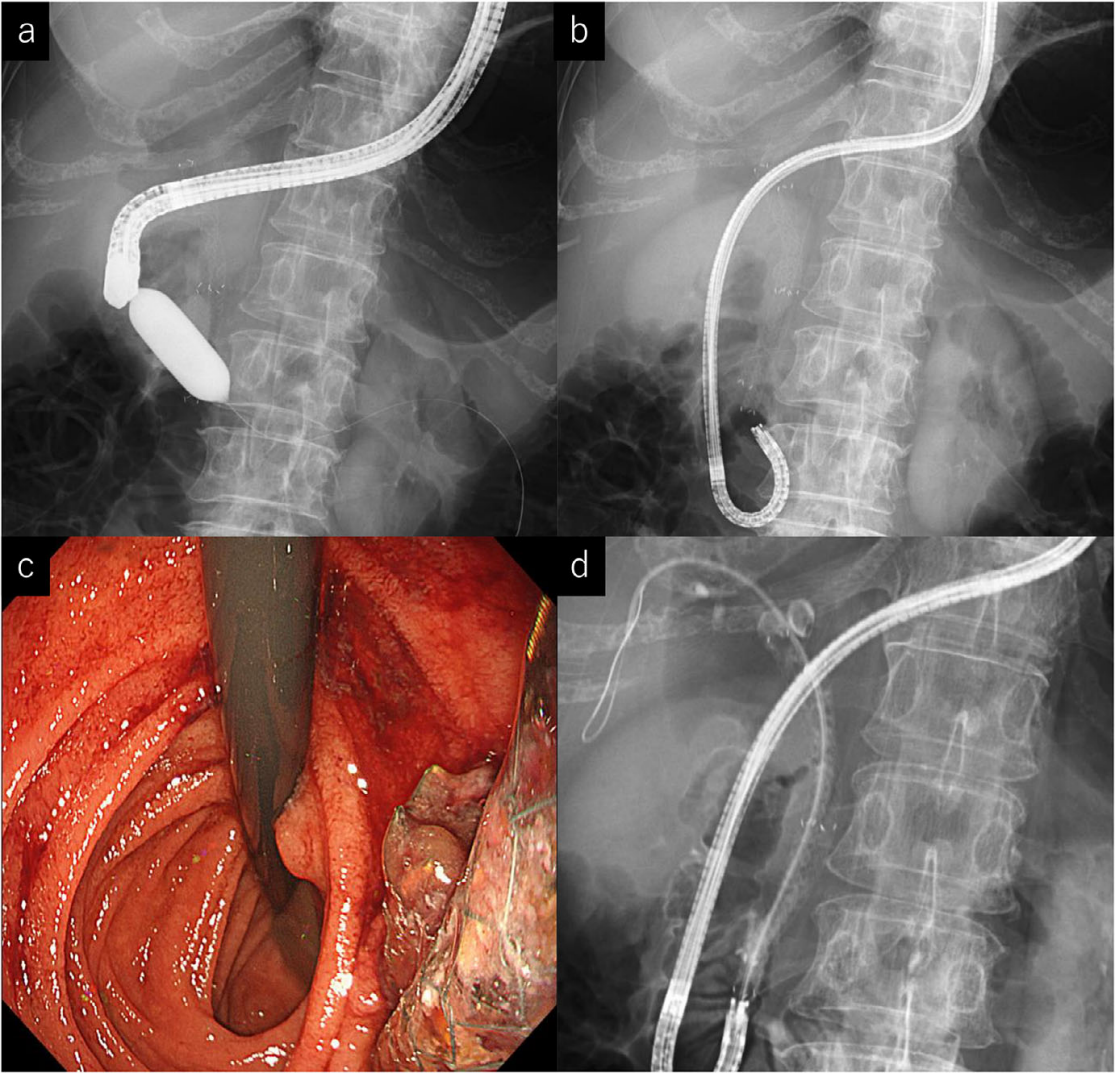
(a) Despite the use of the balloon anchor technique, the duodenoscope could not be advanced into the second portion of the duodenum. (b, c) An ultrathin endoscope was advanced into the second portion of the duodenum and retroflexed to visualize the papilla (b: fluoroscopic image, c: endoscopic image). (d) A 5‐Fr ERBD tube was placed through the MHSEMSs. ERBD, endoscopic retrograde biliary drainage; MHSEMSs, multi‐hole self‐expandable metal stents.

Second intervention: Several months later, she developed cholangitis due to stent occlusion. Repeated ERCP using guidewire‐assisted and balloon‐anchor techniques (Figure 2a) failed. Because of the prior left hepatectomy, EUS‐hepaticogastrostomy (HGS) was not possible. An ultrathin endoscope (GIF‐1200N; Olympus, Figure S1b) was advanced into the second portion of the duodenum, retroflexed to visualize the papilla (Figure 2b,c), and a 5Fr‐endoscopic retrograde biliary drainage (ERBD) tube (10cm; Medi‐Globe GmbH, Rohrdorf, Germany) was placed through the MHSEMS (Figure 2d). However, cholangitis recurred shortly, complicated by multiple hepatic abscesses (Figure S1c).

**FIGURE 3:**
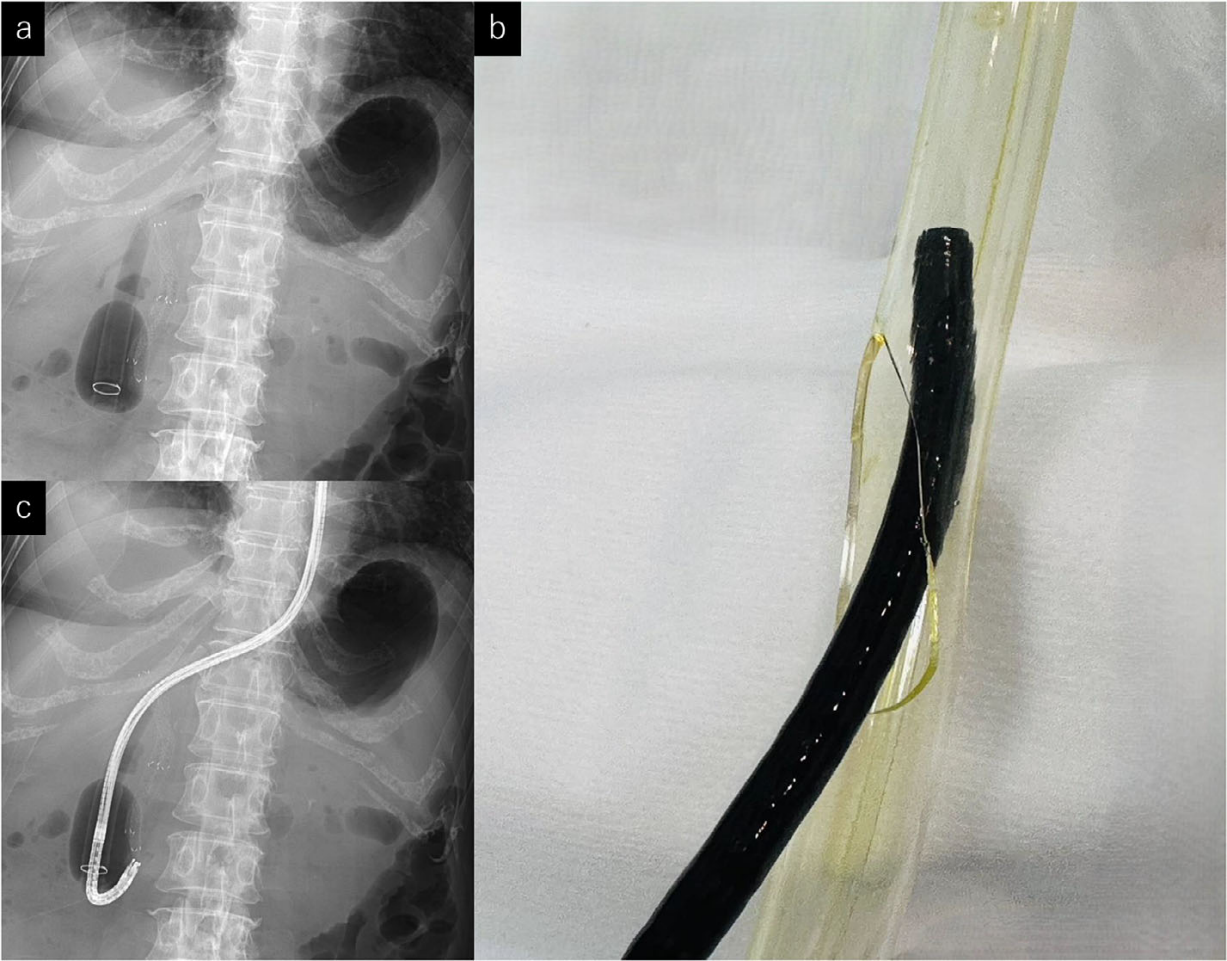
(a) A DBE was advanced to the second portion of the duodenum, and only the overtube was left in place. DBE: double‐balloon enteroscope. (b) A side hole was created in the overtube approximately 10 cm distal to the insertion port, enabling passage of an ultrathin endoscope. (c) The ultrathin endoscope was advanced through the side hole into the duodenum, retroflexed, and successfully visualized the papilla.

**FIGURE 4:**
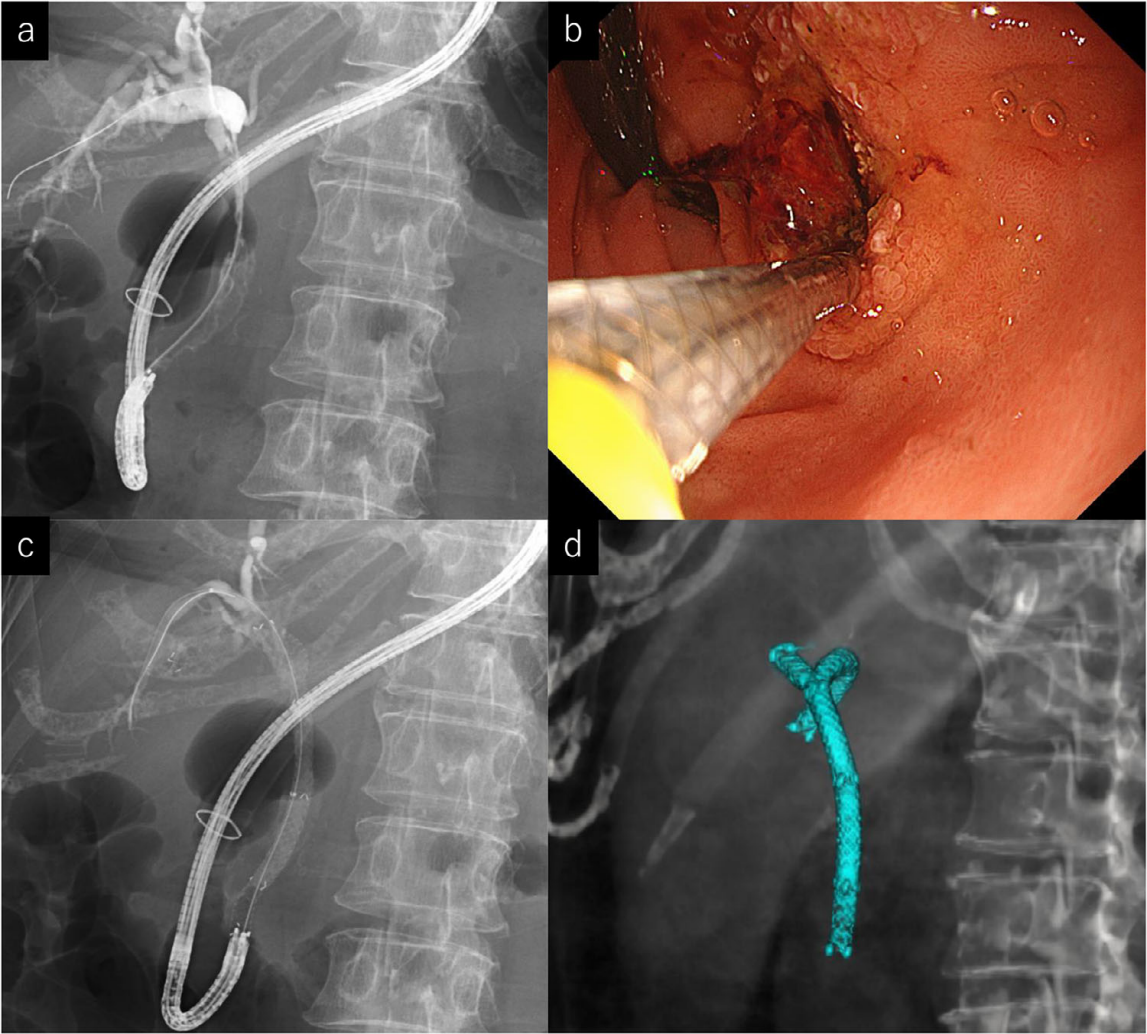
(a) Using the overtube‐assisted ultrathin endoscope approach, cholangiography demonstrated hilar biliary stricture. (b, c) With retroflexion of the ultrathin endoscope, MHSEMSs were deployed into the right anterior and posterior segmental bile ducts using the partial stent‐in‐stent technique. MHSEMSs: multi‐hole self‐expandable metal stents. (d) Three‐dimensional reconstruction of the stents based on CT images after stent placement. CT, computed tomography.

Third intervention with overtube modification: Subsequent attempts to reinsert the ultrathin endoscope failed due to worsening adhesions (Figure S1d). A forward‐viewing scope (GIF‐EZ1500, Olympus/ EI‐580BT; FUJIFILM, Tokyo, Japan) could reach the duodenum, but retroflexion caused resistance and was deemed unsafe because of perforation risk. We therefore advanced a DBE overtube (TS‐13101; FUJIFILM) to the duodenum and withdrew the scope (Figure 3a). However, due to the length of the overtube, conventional insertion of the ultrathin endoscope through the proximal opening did not provide sufficient length to achieve retroflexion in the duodenum. A side hole was therefore created in the overtube approximately 10 cm distal to the insertion port, enabling passage of an ultrathin endoscope (Figure 3b). Through this side hole, the ultrathin endoscope was advanced into the duodenum, retroflexed, and successfully visualized the papilla (Figure 3c). After exchanging the scope for an EI‐580BT, the previously placed MHSEMSs were removed in a downward viewing position through the scope using a snare (Snare Master Plus; Olympus). The ultrathin endoscope was reinserted, and cholangiography confirmed a hilar stricture (Figure 4a). Although side‐by‐side stenting was planned, the MHSEMS delivery system could not pass in parallel. Instead, a partial stent‐in‐stent technique was performed, successfully deploying MHSEMSs into the right anterior (6 mm×10 cm) and posterior (6 mm×12 cm) segmental bile ducts (Figure 4b–d). The patient's cholangitis and hepatic abscess improved, allowing resumption of chemotherapy.

## 3 Discussion

This case illustrates several important points. Conventional ERCP can fail in patients with severe anatomical changes due to prior surgery or tumor spread. Techniques such as guidewire assistance or balloon anchoring [3, 4] may sometimes enable access, but these strategies are not universally successful. In our patient, such approaches allowed the initial metal stent placement but became ineffective as peritoneal dissemination and adhesions progressed. Although EUS‐BD is a reliable alternative [2, 5], some conditions preclude its use. In particular, after left hepatectomy, the puncture route for hepaticogastrostomy may not be available. Even when technically feasible, EUS‐BD carries risks of bile leakage (peritonitis) [6, 7] or stent migration [1], which may be critical in fragile oncology patients. Thus, reliance solely on EUS‐BD is not always appropriate, and other strategies should be considered. We demonstrated a novel solution using a DBE overtube modified with a side hole. The overtube's original length prevented retroflexion of the ultrathin endoscope. By creating a side hole approximately 10 cm distal to the insertion port, we secured a working length that allowed stable retroflexion in the duodenum. This modification enabled safe scope maneuverability and direct access to the papilla. To our knowledge, no previous reports have described this method. The development of MHSEMS with a thin 5.9‐Fr delivery system enabled compatibility with the 2.2‐mm channel of the ultrathin endoscope, allowing successful stent deployment. Previously, SEMSs required larger channels (>3.2 mm), limiting ultrathin endoscopes. This advance expanded their role from diagnostic to therapeutic use. In our case, the side holes of the MHSEMS facilitated a partial stent‐in‐stent technique, allowing deployment of an additional stent through the previously placed device, contributing to the success of re‐intervention. This approach offered an alternative to PTBD, which is invasive and negatively impacts quality of life. Oncology patients in particular may benefit from internal drainage, which reduces infection risk and allows continuation of chemotherapy.

Beyond this individual case, several implications deserve mention. The combination of ultrathin endoscopes and thin delivery systems suggests new possibilities for therapeutic interventions in anatomically difficult cases. The adaptation of the DBE overtube demonstrates how existing devices can be modified to extend endoscopic options. Previous reports have demonstrated overtube (ST‐CB1; Olympus)‐assisted advancement of a side‐viewing duodenoscope in patients with gastric deformity or cascade stomach [8, 9]. While these techniques are useful, they have procedural limitations. The duodenoscope must first be inserted into the overtube without a top hood, and the hood is mounted afterward before advancing into the patient. Once the overtube remains in place, the duodenoscope cannot be reinserted, restricting repeated insertions or scope exchanges. In contrast, overtube insertion under direct visualization of flexures and strictures with a forward‐viewing endoscope ensures safer and more controlled advancement, offering a distinct procedural advantage. Our method thus provides a practical and safer alternative, particularly in institutions equipped only with DBE systems. However, the use of a side‐viewing duodenoscope allows greater familiarity and broader therapeutic options, whereas procedures using an ultrathin endoscope remain technically challenging due to limited channel size and maneuverability. Therefore, treatment strategies should be selected according to individual case characteristics.

The safety of this approach must also be considered. Creating a side hole may compromise overtube integrity, and repeated retroflexion carries a risk of perforation. Although Hirata et al. have described the safety of retroflex position using forward‐viewing endoscopes [10], it is crucial to select treatment strategies with careful consideration of potential risks in each individual case. This method should be reserved for expert centers with surgical backup. By combining an overtube modification, an ultrathin endoscope, and slim MHSEMS delivery systems, we achieved effective biliary drainage when ERCP and EUS‐BD were not feasible. We report the first case of biliary drainage using a novel overtube‐assisted ultrathin endoscopic technique with MHSEMS deployment. This novel overtube‐assisted ultrathin endoscopic technique may expand therapeutic options for selected patients with challenging biliary obstruction.

## Author Contributions

**Akinobu Koiwai**: prepared the first draft of the manuscript. **Akinobu Koiwai, Morihisa Hirota, Kei Ishikawa, Chihiro Yunomura, Takuro Nakaya, Yuki Miyashita,** and **Nana Inomata**: managed the patient. **Morihisa Hirota** and **Kennichi Satoh**: revised the manuscript. All authors approved the final version of the manuscript.

## Funding

The authors received no specific funding for this work.

## Conflicts of Interest

The authors declare no conflicts of interest.

## Supporting information




**Supplementary Figure 1**: (a) Structure of the MHSEMS (HANAROSTENT Biliary Multi Hole Benefit; Boston Scientific, MA, USA). The MHSEMS is a fully covered metallic stent designed with multiple longitudinally aligned side holes arranged in alternating rows around the circumference of the stent. These side holes maintain bile flow through collateral channels, even when the main lumen is partially occluded, and also facilitate guidewire passage during reintervention. MHSEMS: multi‐hole self‐expandable metal stent. (b) Ultrathin endoscope (GIF‐1200N; Olympus, Tokyo, Japan). The GIF‐1200N is an ultrathin endoscope with an outer diameter of 5.4 mm and a working channel of 2.2 mm, originally designed for trans‐nasal endoscopy. (c) CECT revealed multiple small hepatic abscesses, presumed to be due to cholangitis. CECT: contrast‐enhanced computed tomography. Fluoroscopic image showing failed advancement of the ultrathin endoscope into the second portion of the duodenum, despite guidewire‐assisted insertion attempts.

## References

[1] H. Isayama , Y. Nakai , T. Itoi , et al., “Clinical Practice Guidelines for Safe Performance of Endoscopic Ultrasound/Ultrasonography‐guided Biliary Drainage: 2018,” Journal of Hepato‐Biliary‐Pancreatic Sciences 26 (2019): 249–269. 31025816 10.1002/jhbp.631 PMC7064894

[2] A. Y. B. Teoh , V. Dhir , M. Kida , et al., “Consensus Guidelines on the Optimal Management in Interventional EUS Procedures: Results From the Asian EUS Group RAND/UCLA Expert Panel,” Gut 67 (2018): 1209–1228. 29463614 10.1136/gutjnl-2017-314341

[3] T. Iemoto , Y. Yamada , T. Yoshie , H. Hayashi , T. Abe , and T. Ose , “Large‐balloon Anchor Technique for Endoscopic Retrograde Cholangiopancreatography in a Patient With Esophageal Hiatal Hernia,” Endoscopy 55 (2023): E858–e859. 37433325 10.1055/a-2106-1967 PMC10335859

[4] S. Kawaguchi , T. Ohtsu , R. Itai , S. Terada , S. Endo , and N. Shirane , “Large Balloon Anchor Technique for Endoscopic Retrograde Cholangiopancreatography Required for Esophagogastroduodenal Deformities,” Internal Medicine 60 (2021): 2175–2180. 33612682 10.2169/internalmedicine.6624-20 PMC8355398

[5] S. W. van der Merwe , R. L. J. van Wanrooij , M. Bronswijk , et al., “Therapeutic Endoscopic Ultrasound: European Society of Gastrointestinal Endoscopy (ESGE) Guideline,” Endoscopy 54 (2022): 185–205. 34937098 10.1055/a-1717-1391

[6] K. Bishay , D. Boyne , M. Yaghoobi , et al., “Endoscopic Ultrasound‐guided Transmural Approach versus ERCP‐guided Transpapillary Approach for Primary Decompression of Malignant Biliary Obstruction: A Meta‐analysis,” Endoscopy 51 (2019): 950–960. 31121627 10.1055/a-0901-7343

[7] L. Poincloux , O. Rouquette , E. Buc , et al., “Endoscopic Ultrasound‐guided Biliary Drainage After Failed ERCP: Cumulative Experience of 101 Procedures at a Single Center,” Endoscopy 47 (2015): 794–801. 25961443 10.1055/s-0034-1391988

[8] H. Saito , Y. Kadowaki , A. Fujimoto , K. Ohmoto , and S. Tada , “Side‐viewing Scope Insertion Using a Large‐diameter Overtube Designed for Colonoscopy in a Patient With a Cascade Stomach,” Clinical Endoscopy 56 (2023): 256–257. 36600658 10.5946/ce.2022.089 PMC10073850

[9] Y. Ohashi , T. Iwashita , S. Iwata , S. Uemura , and M. Shimizu , “Facilitating Duodenoscope Insertion With a Balloon Overtube in a Patient With Gastric Deformity,” Endoscopy 57 (2025): E365–E366. 40328327 10.1055/a-2584-1901 PMC12055410

[10] Y. Hirata , K. Iida , K. Takahashi , et al., “Transpapillary Biliary Drainage Using a Forward‐viewing Endoscope for Patients With Distal Malignant Biliary Obstruction and Type I Duodenal Stenosis,” Endoscopy International Open 13 (2025): a25542784. 40230561 10.1055/a-2554-2784 PMC11996015

