# Supplementary figures and images for: A Novel Technique of Overtube‐assisted Ultrathin Endoscopic Biliary Drainage Using Multi‐Hole Self‐Expandable Metal Stents: A Case Report
Source: DEN Open. 2025 Nov 26;6(1):e70253. doi: 10.1002/deo2.70253 (PMC12649053; doi:10.1002/deo2.70253)

## Slide 1
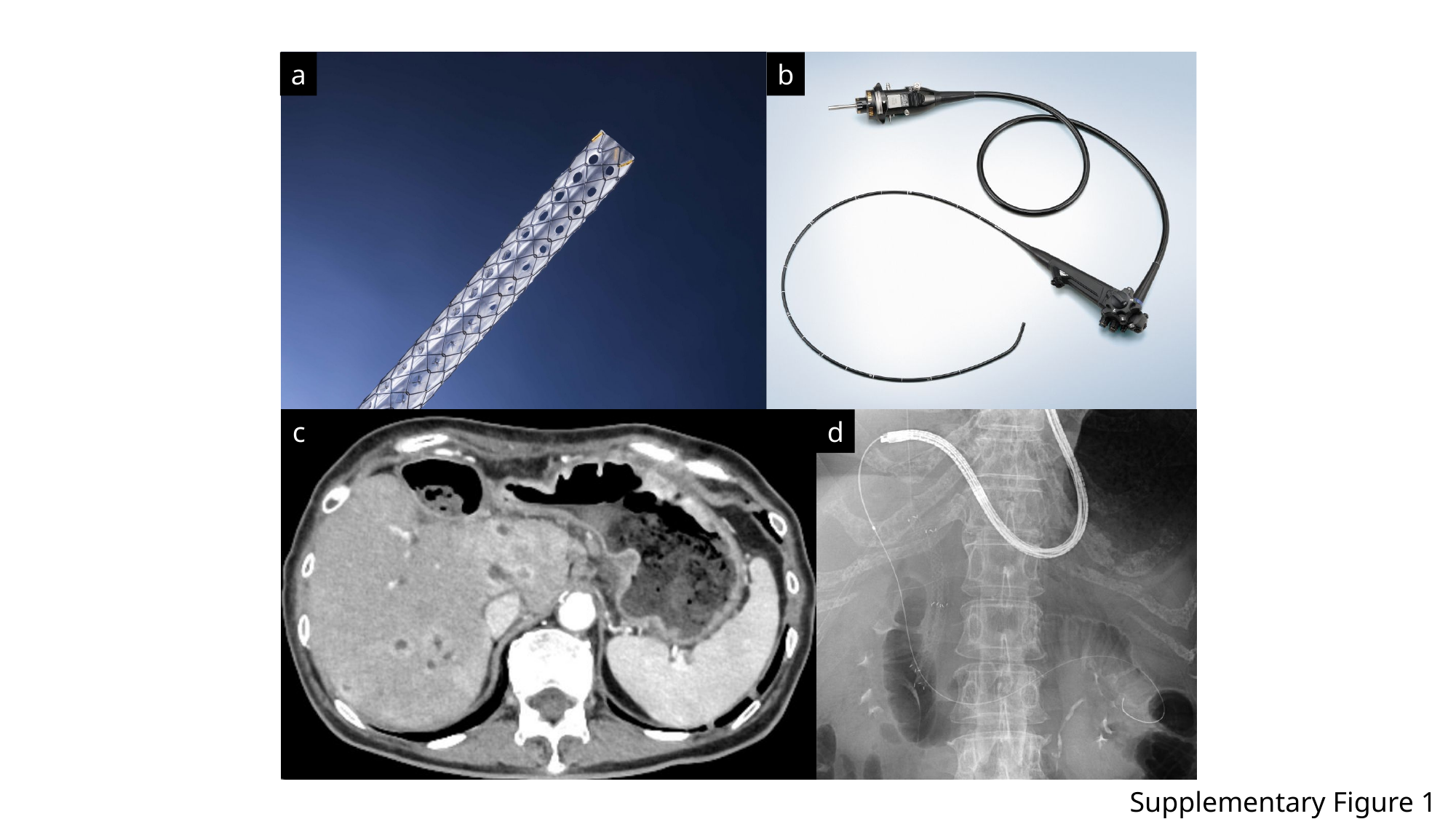

a
b
c
d
Supplementary Figure 1

Supplement: Supplementary file 1 — Supplementary Figure 1: (a) Structure of the MHSEMS (HANAROSTENT Biliary Multi Hole Benefit; Boston Scientific, MA, USA). The MHSEMS is a fully covered metallic stent designed with multiple longitudinally aligned side holes arranged in alternating rows around the circumference of the stent. These side holes maintain bile flow through collateral channels, even when the main lumen is partially occluded, and also facilitate guidewire passage during reintervention. MHSEMS: multi‐hole self‐expandable metal stent. (b) Ultrathin endoscope (GIF‐1200N; Olympus, Tokyo, Japan). The GIF‐1200N is an ultrathin endoscope with an outer diameter of 5.4 mm and a working channel of 2.2 mm, originally designed for trans‐nasal endoscopy. (c) CECT revealed multiple small hepatic abscesses, presumed to be due to cholangitis. CECT: contrast‐enhanced computed tomography. Fluoroscopic image showing failed advancement of the ultrathin endoscope into the second portion of the duodenum, despite guidewire‐assisted insertion attempts. [file DEO2-6-e70253-s001.pptx]
